# Magnetic Bead Handling Using a Paper-Based Device for Quantitative Point-of-Care Testing

**DOI:** 10.3390/bios12090680

**Published:** 2022-08-25

**Authors:** Kevin Arias-Alpízar, Ana Sánchez-Cano, Judit Prat-Trunas, Elena Sulleiro, Pau Bosch-Nicolau, Fernando Salvador, Inés Oliveira, Israel Molina, Adrián Sánchez-Montalvá, Eva Baldrich

**Affiliations:** 1 Diagnostic Nanotools Group, Vall d’Hebron Institut de Recerca (VHIR), 08035 Barcelona, Spain; 2 Universitat Autònoma de Barcelona (UAB), 08193 Barcelona, Spain; 3 International Health Unit Vall d’Hebron-Drassanes, Vall d’Hebron Hospital Universitari, PROSICS Barcelona, 08035 Barcelona, Spain; 4 Centro de Investigación Biomédica en Red de Enfermedades Infecciosas (CIBERINFEC), Instituto de Salud Carlos III, 28028 Madrid, Spain

**Keywords:** immuno-modified magnetic beads, paper-based diagnostic device, low-cost assay automation, smartphone colorimetric detection, point-of-care testing, malaria quantitative diagnosis

## Abstract

Microfluidic paper-based analytical devices (μPADs) have been extensively proposed as ideal tools for point-of-care (POC) testing with minimal user training and technical requirements. However, most μPADs use dried bioreagents, which complicate production, reduce device reproducibility and stability, and require transport and storage under temperature and humidity-controlled conditions. In this work, we propose a μPAD produced using an affordable craft-cutter and stored at room temperature, which is used to partially automate a single-step colorimetric magneto-immunoassay. As a proof-of-concept, the μPAD has been applied to the quantitative detection of *Plasmodium falciparum* lactate dehydrogenase (Pf-LDH), a biomarker of malaria infection. In this system, detection is based on a single-step magneto-immunoassay that consists of a single 5-min incubation of the lysed blood sample with immuno-modified magnetic beads (MB), detection antibody, and an enzymatic signal amplifier (Poly-HRP). This mixture is then transferred to a single-piece paper device where, after on-chip MB magnetic concentration and washing, signal generation is achieved by adding a chromogenic enzyme substrate. The colorimetric readout is achieved by the naked eye or using a smartphone camera and free software for image analysis. This μPAD afforded quantitative Pf-LDH detection in <15 min, with a detection limit of 6.25 ng mL^−1^ when the result was interpreted by the naked eye and 1.4 ng mL^−1^ when analysed using the smartphone imaging system. Moreover, the study of a battery of clinical samples revealed concentrations of Pf-LDH that correlated with those provided by the reference ELISA and with better sensitivity than a commercial rapid diagnostic test (RDT). These results demonstrate that magneto-immunoassays can be partly automated by employing a μPAD, achieving a level of handling that approaches the requirements of POC testing.

## 1. Introduction

Paper and paper-like materials are extensively employed to produce analytical tools [1,2]. Compared to other alternatives, such as glass-, silicon- or polymer-based chips, μPADs are inexpensive, relatively easy to fabricate, and convenient to dispose of. The porous membrane provides filtration and pre-treatment of complex samples, large surface areas for reagent incorporation, and passive solution pumping without external power sources or equipment. When coupled to the visual interpretation of a colorimetric readout, μPADs approach ideal POC diagnostic devices, matching the ASSURED criteria established by the World Health Organization (WHO): affordable, sensitive, specific, user-friendly, rapid and robust, equipment free, and deliverable to end-users [3].

The most widely used μPADs are lateral-flow assays (LFAs), which consist of 4 types of overlapping membranes (sample, conjugate, test and absorbent pads, respectively), two of them containing dried reagents [4]. To carry a test, the user dispenses the sample (and sometimes running buffer), which wicks along the device to interact with a labelled detection antibody (d-Ab) and the immobilized capture antibody (c-Ab), forming coloured bands that are interpreted visually or using hand-held readers [5]. More sophisticated μPADs incorporate hydrophobic barriers to define hydrophilic channels and chambers (such as by paper patterning, multi-component stacking, or folding into three-dimensional origami chips) [1,2]. When reagent-modified, μPADs display limited stability and should be distributed and stored under controlled temperature and humidity [2]. In addition, reagent-modified μPADs can be used only to detect the analyte they were produced for.

Malaria is a disease caused by *Plasmodium* parasites that are transmitted to humans by the bite of infected female *Anopheles* mosquitoes [6]. Despite being curable, malaria took the life of 627,000 people and caused 241 million cases in 2020 [7]. Of the human-infecting *Plasmodium* species, *P. falciparum* is the most frequent and deadly, accounting for 94% of the cases and deaths reported worldwide. There is a general agreement that factors such as increasing parasite drug resistance [8], migration processes [9], climate change effects [10], growth of the population at risk, and the COVID-19 pandemic [8] combine to jeopardise the advances made over the last decades to control the disease in the endemic countries, increasing the risk of malaria reintroduction in areas from which it had been eliminated.

Currently, the “gold standard” for the diagnosis of malaria is microscopy [11,12]. Although a well-trained microscopist can detect down to 50–100 parasites per μL of blood (parasite μL^−1^) under typical field conditions [13], microscopy relies on expertise and subjective result interpretation, requires >1 h per sample, and cannot detect low parasitemias (so-called submicroscopic malaria). In centralised laboratories, molecular techniques, such as the polymerase chain reaction (PCR), are gradually being incorporated, achieving exceptional LODs (around 1 parasite μL^−1^), often in fully automated and species-specific assay formats. However, PCR demands long analysis time, experienced personnel, sophisticated infrastructure and high operational costs. Finally, RDTs have been positioned as fast, inexpensive, and easy-to-use tools for malaria POC testing in low/middle-resource settings (such as the countries where malaria is more prevalent) or as the first test in high-resource non-endemic settings before performing confirmatory analysis [14,15,16]. Most RDTs are LFA-based devices that detect *Plasmodium* antigens in a drop of blood, in <30 min, at a cost of 1–5 € [17]. Nonetheless, RDTs display limitations as well, including variable performance depending on the storage and environmental conditions, subjective result interpretation, qualitative (yes/no) response, insufficient sensitivity, and limited detection of “non-*falciparum*” species [18,19].

Attempts have been made to produce upgraded µPADs. For instance, Pilon dos Santos et al. produced a µPAD to detect *P. falciparum* histidine-rich protein 2 (Pf-HRP2) [20]. The paper chip, cut using a CO_2_ laser plotter, displayed 3 discrete areas separated by narrow microfluidic channels. The first served for the addition of sample and peroxidase-labelled d-Ab; the second displayed immobilised c-Ab; and the third granted solution pumping and waste storage. The system displayed an LOD of 4.5–5.0 ng mL^−1^ of Pf-HRP2 in saline solution (equivalent to 59–65 parasites μL^−1^) but was not tested in clinical samples. Since Pf-HRP2 is only present in *P. falciparum* and deletions in the pfhrp2/3 genes may prevent detection by HRP2-based RDTs, alternative Pan *Plasmodium* biomarkers have been targeted [21,22,23,24]. A multiplexed µPADs was produced by Deraney and co-workers by stacking 8 layers of wax-patterned paper, two of them modified with gold-labelled d-Ab and immobilised c-Ab [25]. The device detected Pf-HRP2 and Pan *Plasmodium* lactate dehydrogenase (pLDH) in about 20 min, with LODs of 20.3 ng mL^−1^ and 80.2 ng mL^−1^, respectively, and achieved detection in spiked lysed blood. Singh et al. employed aptamer-coated MB to trap pLDH and *P. falciparum* glutamate dehydrogenase (Pf-GDH), which were then incubated in a chromogenic enzyme substrate solution [26]. The supernatant was next transferred to a piece of chemically modified chromatographic paper for colour interpretation by the naked eye (qualitative) or using image analysis (quantitative), achieving LODs down to 68–69 pM in spiked serum in about 95 min (equivalent to 2.3–2.4 ng mL^−1^). The electrochemical µPAD reported by Ruiz-Vega and co-workers included a sample filtration unit, a double-sided screen-printed paper electrode and absorbent pads in a low-cost magnetic holder [27]. The system facilitated the partial automation of a single-step magneto-immunoassay and detected Pf-LDH in lysed whole blood. Pf-LDH quantitation was afforded down to 2.47 ng mL^−1^ in about 20 min, identifying patients presenting malaria parasitemias > 0.3%.

Here, we develop a µPAD for magneto-immunoassay POC operation. As a proof-of-concept, we report the fast and quantitative detection of Pf-LDH based on a single-step magneto-immunoassay, a generic single-piece microfluidic paper device and smartphone-based colorimetry. The system affords quantitative Pf-LDH detection in lysed whole blood samples in <20 min, with an LOD of 1.4 ng mL^−1^ and minor user intervention. However, the storage-stable paper device could be employed to detect other analytes by just adjusting the magneto-immunoassay reagents.

## 2. Material and Methods

### 2.1. Reagents and Biocomponents

Recombinant Pf-LDH was provided by CTK Biotech (San Diego, CA, USA). Monoclonal capture and detection antibodies to pLDH (C01834M and C01835M, c-MAb and d-MAb, respectively) were from Meridian Bioscience (Memphis, TE, USA). The latter were modified with biotin to produce biotinylated d-MAb (bd-MAb; Supporting Information). Carboxylic acid MB of 1 μm diameter (Dynabeads MyOne, Invitrogen Ref. 65011 and SpeedBead Magnetic Carboxylate, GEHealthcare Ref. 45152105050250), Streptavidin Poly-HRP (Ref. 21140), sulphuric acid 1 M and 1-ethyl-3-(3-dimethylaminopropyl) carbodiimide hydrochloride (EDC) were from Thermo Fisher Scientific (Barcelona, Spain). Bovine serum albumin (BSA), Triton X-100, Tween 20, 3,3′,5,5′-Tetramethylbenzidine Liquid Substrate System (TMB; Ref. T0440), and 2-(N-morpholino)ethanesulfonic acid hydrate (MES) were from Sigma-Aldrich (Madrid, Spain). Phosphate-buffered saline tablets (PBS; Barcelona, Spain) produced KH_2_PO_4_ 250 mM sodium phosphate, NaCl 150 mM, and KCl 2.7 mM, pH 7.4. Reagent Diluent (10 × RD, Ref. DY995; equivalent to 10 × PBS, 10% BSA) was from R&D (Abingdon, UK). The membranes used for device production were Standard 17 and CF5 (Refs. 17114594 and 29008181; Cytiva Europe, Freiburg, Germany). For washing, PBS was supplemented with 0.05% of Tween 20 (PBS-T). Unless otherwise stated, blocking and incubation steps were carried out with PBS supplemented with BSA 1% and 0.05% of Tween 20 (PBST-BSA).

### 2.2. Device Fabrication

**Figure 1:**
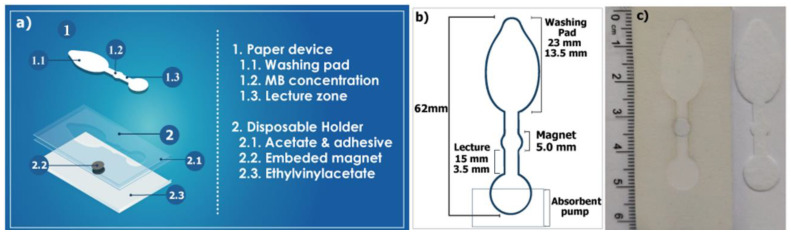
(**a**) Schematic representation of the system developed, which resembled that described in [28] but displayed different geometric features. (**b**) Dimensions of the single-piece paper device. (**c**) Picture of the reusable magnetic holder (**left**) and the disposable paper device produced with Standard 17 membrane (**right**).

The system for partial magneto-immunoassay operation and detection automation included two components, a disposable μPAD and a reusable magnetic holder, which were assembled as previously described in [28] (Figure 1a). Briefly, the main body of the μPAD was a single-piece device, which was designed using Silhouette Studio version 4.4.476 and cut on Standard 17 using an inexpensive craft plotter, Silhouette Cameo 3 (Silhouette America, Lehi, UT, USA; Figure 1b,c). It contained three distinguishable sections (Figure 1b). The first one was a tear-shaped washing pad (23 mm × 13.5 mm) for the addition of sample and assay reagents. Accordingly, this section was designed to display high-volume load capacity and efficient solution flow towards the second section. This was an MB concentration zone 5 mm in diameter, which was placed onto a magnet when the paper device was positioned in the magnetic holder. Apart from MB retention, this section also provided a lecture zone for the colorimetric readout. Finally, the third section was a circular end. This was allocated below an absorbent pad (26 × 16 mm), which was produced using a guillotine and CF5. This pad functioned as a flow-driving absorbent pump and a waste storage unit.

On the other hand, the magnetic holder was a reusable device made of a first layer of ethylene-vinyl acetate (purchased from a local retailer). It displayed a pit that was made with a biopsy punch and accommodated a neodymium magnet (1 mm thick, 5 mm diameter). Two layers of acetate, carved with the Silhouette, were secured on top with double-sided adhesive. The bottom one was plain to keep the magnet in place and prevent direct contact with the MBs. The other displayed a cavity to accommodate the paper device and guarantee the correct alignment with the magnet. Once placed in the holder, the paper device fit in this cavity and sat directly onto the first acetate.

Paper devices were blocked by immersion in BSA 5%, Tween 0.5% for 15 min at room temperature, and were then washed twice with PBS-T for 3 min each. Finally, the devices were rinsed with water, dried for 30 min at 37.5 °C and stored in a ziplock pouch until used.

### 2.3. Smartphone Imaging System

A smartphone-integrated imaging system was designed to take photographs of the μPAD colorimetric readout (Figure S1 in Supplementary Material). This included a homemade dark box produced using a cardboard box (28 × 16 × 10 cm), a dimmable strip of white 6000K LED lights with a power of 1200 lm for lighting the chamber, and a smartphone placed on the lid, which had a hole for the phone camera. For image acquisition, paper devices were removed from the magnetic holders and were placed in the centre of the dark box. The box was closed, the lights were turned on, and images were obtained using the smartphone camera. When processing the images with ImageJ software, the signal at the detection pad was calculated for each paper device by subtracting the background registered for the whole device, converting the image into an 8-bit format and setting the colour threshold to 235 to obtain the mean grey value. Thus, the colorimetric signal was taken using the whole sensor area.

### 2.4. Pf-LDH Magneto-Immunodetection

Unless otherwise stated, MB modified with c-MAb (c-MAb-MB; Supporting information) were incubated in 1.5 mL Eppendorf tubes at 24 °C, protected from light in a thermoshaker (Thermal Shake lite, VWR International, Leuven, Belgium), and washed using a magnetic separator (BILATEST, Merck Life Science, Madrid, Spain).

#### 2.4.1. Magneto-Immunoassay in Tubes (Classical Approach)

The magneto-immunoassay originated from a previous development (Figure S2) [27,28]. Briefly, c-MAb-MB were washed two times with PBS and resuspended in PBST-BSA to a final concentration of 5 mg mL^−1^. Pf-LDH was then agitated at 1500 rpm for 5 min with 4 μL of c-MAb-MB in PBST-BSA supplemented with bd-MAb (75 ng mL^−1^) and Poly-HRP (50 ng mL^−1^) in a final volume of 110 μL. For spectrophotometric assay detection, the c-MAb-MB/Pf-LDH/bd-MAb/Poly-HRP complexes were washed twice with 150 μL of PBST, resuspended in 100 μL of TMB and stirred for 20 min at 1500 rpm. After this, MBs were concentrated, the supernatant was transferred to 96-well plates, and 50 μL of 1 M sulphuric acid was added to stop the reaction. Finally, absorbance was measured at 450 nm using a Sunrise plate reader (Tecan Group, Männedorf, Switzerland).

#### 2.4.2. Magneto-Immunoassay Using the μPAD (POC Approach)

For on-chip MB washing and colorimetric assay detection, after the 5-min immunocapture performed in tubes (as indicated in Section 2.4.1), the mixture of c-MAb-MB/Pf-LDH/bd-MAb/Poly-HRP was directly placed in the μPAD washing pad. Four consecutive washes were next carried out by adding 100 μL of PBST each time on the washing pad. Then, 50 μL of TMB was added to the detection pad, where the magnetic complexes had been retained by a magnet, and the device was incubated for 5 min without agitation at room temperature before image capture.

#### 2.4.3. Pf-LDH Detection in Whole Blood Clinical Samples

Malaria patients and healthy individuals were recruited during the period of 2018–2020 at Vall d’Hebron University Hospital. Peripheral blood samples were obtained in heparin collection tubes before the administration of any antimalarial drugs. Malaria acute infections were confirmed by microscopy and/or PCR. The study was approved by the Ethics Committee of Vall d’Hebron University Hospital (PR(AG)30/2018), and the corresponding informed consent was obtained from all patients.

For detection of Pf-LDH using the μPAD, samples were diluted 1:1 with lysis buffer (50 mM KH_2_PO_4_, 300 mM NaCl, 0.25 M imidazole, 1% Triton X-100) and were incubated for 5 min at room temperature. Samples were diluted 1:10–1:100 with PBST-BSA and were processed as described in Section 2.4.2. All samples were analysed 2 times independently and were assayed in parallel by commercial RDT, microscopy and ELISA [29].

### 2.5. Data Analysis

Paper devices were used just once (i.e., each device for a single Pf-LDH concentration). Except for the detection of clinical samples (n = 2), graphs show the average of the colorimetric signal readout registered for no less than three independent replicates. In the same way, error bars correspond to the standard deviation (SD) of those replicates. The limit of detection (LOD) and the limit of quantification (LOQ) were calculated as the average of the signals registered for the blanks (i.e., experiments carried with all the reagents in the absence of Pf-LDH) plus 3 times and 10 times their SD, respectively. The sensitivity was calculated from the slope of the linear assay range. The variability was analysed in terms of coefficient of variation (% CV = (SD/mean) × 100). Signal-to-noise ratio (S/N) stands for the signal generated by each Pf-LDH concentration divided by the average signal registered for the blanks.

## 3. Results and Discussion

**Figure 2:**
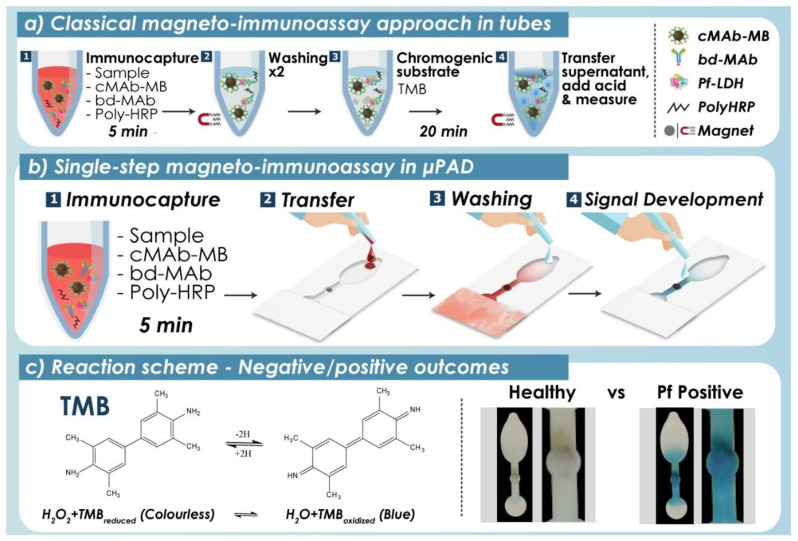
Single-step magneto-immunoassay carried out manually as reported in [28] (**a**) and using the µPAD developed here, which differed in size and shape from that in our previous work to better fit the requirements of colorimetric detection (**b**). (**c**) Scheme of TMB redox reaction (here, Poly-HRP catalyses TMB oxidation coupled to H_2_O_2_ reduction for colour generation), and examples of negative and positive µPAD readouts.

The starting point for this work was a single-step magneto-immunoassay developed previously for Pf-LDH detection [27], with some improvement (Figure 2a and Figure S2) [28]. This consisted of a single 5-min incubation of the sample (lysed whole blood) with a cocktail of three reagents in an Eppendorf tube: cAb-MBs, which allowed fast Pf-LDH capture and concentration from whole blood; bd-MAb to grant binding specificity in a sandwich assay format; and Poly-HRP, an enzymatic signal amplifier formed by streptavidin and hundreds of HRP molecules that provided large signals. In the classical approach, this incubation was followed by two consecutive washing steps with PBS-T. Each washing step entailed placing the tube for 2 min in a magnetic rack for MB concentration, removing the supernatant without disturbing the MB pellet, and resuspending the MBs in the appropriate solution using a pipette. After the last wash, MBs were resuspended in TMB substrate solution, the Poly-HRP enzymatic reaction was allowed to proceed for 20 min, the tube was placed once more in the magnet for 2 min, and the supernatant was transferred to a 96-well plate to quantitate colour intensity. Although fast and efficient in the hands of an expert, this procedure required user training and was incompatible with POC testing carried out by untrained personnel.

The objective of this work was to demonstrate that this type of assay can be partially automated using a simple and inexpensive disposable µPAD (Figure 2b). Here, the colorimetric readout obtained has been interpreted alternatively by semi-quantitative visual inspection or using a smartphone camera and ImageJ free software, which provided quantitative results. Furthermore, this strategy entails the production of a universal paper device, which could be stored and employed to detect alternatively different magneto-immunoassays. The next sections summarize the optimization of the µPAD geometric features, magneto-immunoassay handling using the µPAD, and technology validation with clinical samples.

### 3.1. Production of the µPAD

A µPAD was designed to carry out MB washing on-chip and under flow conditions. This device displayed three sections: a washing pad for the serial addition of the mixture of sample and reagents (c-MAb-MB, poly-HRP and bd-MAb) and the washing buffer; a pad for MB magnetic concentration and colour readout; and the final end with a staked absorbent pad (Figure 1). For its utilization, the µPAD was placed onto a reusable magnetic holder, which consisted of a piece of ethylene-vinyl acetate with an embedded magnet and a plastic cover that facilitated the alignment of the paper device.

**Figure 3:**
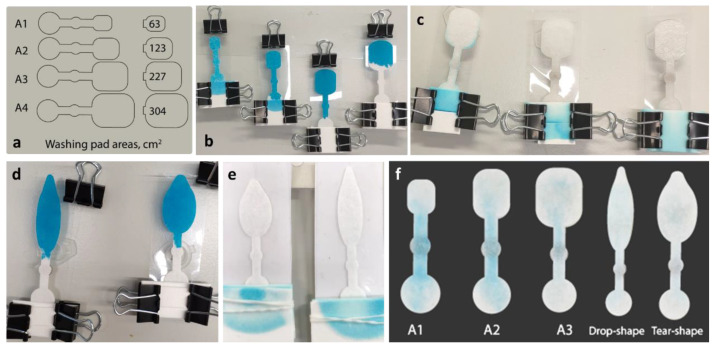
Production of the microfluidic paper device. (**a**) Design of paper devices with washing pads of increasing areas (A1–A4) and (**b**) their absorption capacity after adding MBs in 100 µL of a stained solution. (**c**) Device A3, in which the 100 µL of MB stained solution has been washed away after four consecutive additions of washing buffer of, from left to right, 50, 100, and 200 µL each, while the MBs are retained magnetically in the detection zone (Figure S3). (**d**,**e**) Tear-shaped and drop-shaped devices before (**d**) and after (**e**) the stained solution has been washed away (4 washes, 100 µL each). (**f**) Background noise obtained for the magneto-immunoassay, carried in 100 μL of lysed whole blood (diluted 1:100) after washing 4 times (100 μL each) and adding TMB substrate solution. Pictures of drop- and tear-shaped devices are not to scale.

The µPAD initially displayed a rectangular washing pad with smooth edges in order to facilitate solution flow and limit non-specific biocomponent retention. Figure 3a,b show 4 devices with washing pads of increasing area after adding the MBs in 100 µL of a stained solution, equivalent to the volume of sample and reagents used in the magneto-immunoassay. The two smallest washing pads tested (A1–A2) had a theoretical absorption capacity of 30 and 60 µL and were able to absorb 100 µL of solution only if these were pipetted very slowly, which was not practical. On the other hand, faster pipetting produced solution overflow, with the corresponding loss of MBs. The absorption pad in the A3 device absorbed this volume of solution in about 15 s, soaking the absorption and detection pads completely. In contrast, the largest absorption pad (A4), which had a theoretical adsorption capacity of 125 µL, was not completely wet with this volume of solution, which increased the amount of washing buffer needed later on.

In A3, 3–4 serial additions of 100 µL of washing buffer were enough to wash the 100 µL of stained solution and concentrate the MB in the retention zone (Figure 3c and Figure S3). Lower washing volumes required more additions to accomplish washing and recover the MBs in the detection area, and larger volumes extended the washing steps unnecessarily. In all cases, the peripheral zones of the washing pad were washed less efficiently than the centre (Figure S3). This was concerning because the assay had to be carried out in lysed whole blood, and any unwashed cell debris and reagents could increase the background noise. Accordingly, the washing pad was re-designed to be either drop- or tear-shaped (Figure 3d,e). These two new designs had washing pad areas of around 250 cm^2^ with elongated shapes and very smooth edges to minimise MB dispersion. These geometries granted fast adsorption of the 100 μL of stained solution (Figure 3d), improved the flow of the MBs towards the magnet, and were washed efficiently (Figure 3e).

Finally, the best-performing designs (A1–A3, drop-shaped and tear-shaped) were used to determine MB on-chip washing efficiency. For this, lysed whole blood (Pf-LDH negative) was diluted at 1:100 in PBST-BSA and was incubated with c-MAb-MBs, bd-MAb and Poly-HRP for 5 min, and the mixture was transferred to the device washing pad. Four washes were carried out (100 μL each), the adsorption pads were removed, and TMB was added to the detection area (Figure 3f). Blue colour, attributed to poor washing, formed mainly in the vicinity of the lecture zone, with a significant decrease in colour intensity and dispersion as the washing pad area increased. The lowest background noise was observed for the drop and tear-shaped designs. This indicated that these washing pads provided the best MB washing and removal of leftover components.

### 3.2. Magneto-Immunoassay Handling and Detection Using the µPAD

The operation of the magneto-immunoassay using the µPAD is illustrated in Figure 2b. The assay started with a single 5-min incubation of the sample/Pf-LDH and reagents (c-MAb-MB, poly-HRP and bd-MAb), which was carried out in a tube. The mixture was then transferred to the washing pad of the paper device. Washing buffer was added, which pushed the sample and reagents across the retention zone, where the MBs (and thus cMAb-MB/Pf-LDH/bd-MAb/Poly-HRP complexes in positive samples) were trapped magnetically. This stage was crucial because the washing buffer had to eliminate unbound reagents and non-targeted blood components, directing them towards the absorption pad in order to minimize background noise. Finally, 50 µL of TMB enzyme substrate solution was added to the detection zone, and the device was incubated for 5 min in the dark for colour development. The formation of a blue product was indicative of the presence of Pf-LDH, and the colour intensity was proportional to the concentration of Pf-LDH.

The optimization of magneto-immunoassay performance under flow conditions included the study of the amount and location of TMB addition (Figure S4) and the concentration and type of c-MAb-MBs (Figures S5 and S6).

Dispensing the TMB in the centre of the detection zone directly onto the MBs produced MB random rearrangement, which reduced signal generation and reproducibility (Figure S4a). The highest colour intensity and reproducibility were achieved if the substrate solution was dispensed upstream from the magnet location in the stem that separated the washing and detection areas. Colour intensity also increased with the amount of TMB dispensed (30, 50 and 100 μL), but both in the positive and the negative controls (background noise; Figure S4b). Therefore, the best S/N was achieved using 50 μL of TMB (Figure S4). The amount of MBs used per sample was next decreased in an attempt to reduce the background noise, but the signals and S/N registered in the presence of Pf-LDH decreased as well, as did the assay sensitivity (slope) (Figure S5). A concentration of 20 μg of c-MAb-MB per sample was selected for subsequent experiments. Invitrogen Dynabeads were tentatively substituted by GE SeraMag MB, which, according to the provider, display higher magnetic content. Although these exhibited faster magnetic concentration, they also provided slightly higher background noise and lower signals for all the concentrations of Pf-LDH studied. Accordingly, work continued with Dynabeads, which displayed higher signals for the Pf-LDH positive controls, lower background noise, and higher S/N in the magneto-immunoassay (Figure S6).

### 3.3. Quantitative Detection of Pf-LDH Using a Smartphone Detection System

**Figure 4:**
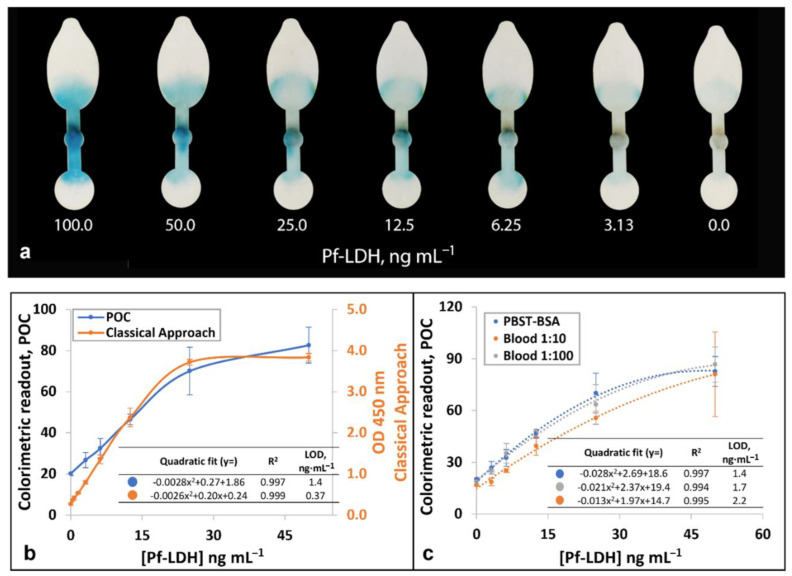
Signals registered in the paper-based magneto-immunoassay for different concentrations of Pf-LDH. (**a**) Photographs of the µPADs and results of semi-quantitative interpretation (Pf-LDH in PBST-BSA). (**b**) Signals obtained for the magneto-immunoassay carried in tubes using an ELISA reader (classical approach) and the µPAD using a smartphone and ImageJ (n = 4; Figure S7). (**c**) Signals registered in the µPAD for Pf-LDH spiked in PBST-BSA or lysed blood (1:10 and 1:100 in PBST-BSA).

Upon optimization, the paper-based magneto-immunoassay took less than 13 min, including single-step immunocapture, washing and detection. Colour intensity was proportional to Pf-LDH concentration between 3.13 ng mL^−1^ and 100 ng mL^−1^, with an LOD of 6 ng mL^−1^ (Figure 4a and Figure S7), demonstrating the feasibility of carrying a semi-quantitative naked eye evaluation. The following step was implementing objective data acquisition and analysis using a smartphone.

Smartphone-based densitometry can be challenging for POC applications due to the difficulty of controlling the positioning of the camera and compensating for the variable background noise. Accordingly, controlling a number of parameters, such as distance and angle between the camera and the paper device, light intensity, and area measured, is key to producing reproducible images and data. Here, an inexpensive dark box was produced for image acquisition using a cardboard box (28 × 16 × 10 cm), modified with a strip of LEDs, used for lighting the chamber and controlling light exposure (Figure S1). A hole (2 × 2 cm) in the lid facilitated the correct positioning of any smartphone camera. Finally, pictures were processed using ImageJ, an open-source image analysis software that allowed subtracting the background noise before calculating colour intensity.

Image analysis of the results obtained in the µPAD for increasing concentrations of Pf-LDH (in PBST-BSA; Figure 4a and Figure S7) allowed producing a calibration plot (Figure 4b). Compared to the assay in tubes, the smartphone-based µPAD produced higher background noise, attributed to less efficient MB washing, which resulted in higher LOD (0.37 ng mL^−1^ in the assay in tubes; 1.4 ng mL^−1^ in the µPAD). Signal saturation was attained at a higher Pf-LDH concentration (50 instead of 25 ng mL^−1^), with variability between independent replicates below 18% (compared to <10% in the classical approach).

A similar experiment was performed on spiked blood. For this, whole lysed blood from healthy individuals was diluted at 1:10 and 1:100 with PBST-BSA and spiked with 0–100 ng·mL^−1^ of Pf-LDH. Finally, it was analysed using the smartphone-based µPAD. As shown in Figure S7, blood was washed away efficiently, and the devices did not display reddish blood leftovers. Furthermore, the calibration plots obtained in PBST-BSA and spiked blood (diluted 1:10 and 1:100) displayed comparable trends, with a linear response between 3.15 ng mL^−1^ and 25 ng mL^−1^ of Pf-LDH concentration (R^2^ > 0.99) and signal saturation around 50 ng mL^−1^ (Figure 4c). In addition, the LODs were, in all cases, below 2.2 ng mL^−1^ of Pf-LDH. Considering that 1 parasite µL^−1^ corresponds to approximately 0.2 ng mL^−1^ of Pf-LDH [29], the LOD was under 11 parasites µL^−1^. This was equivalent to 110 parasites µL^−1^ in prediluted samples when testing blood at 1:10, which is below the threshold value of 200 parasites µL^−1^ recommended by WHO for RDTs [13].

### 3.4. Analysis of Clinical Samples

**Figure 5:**
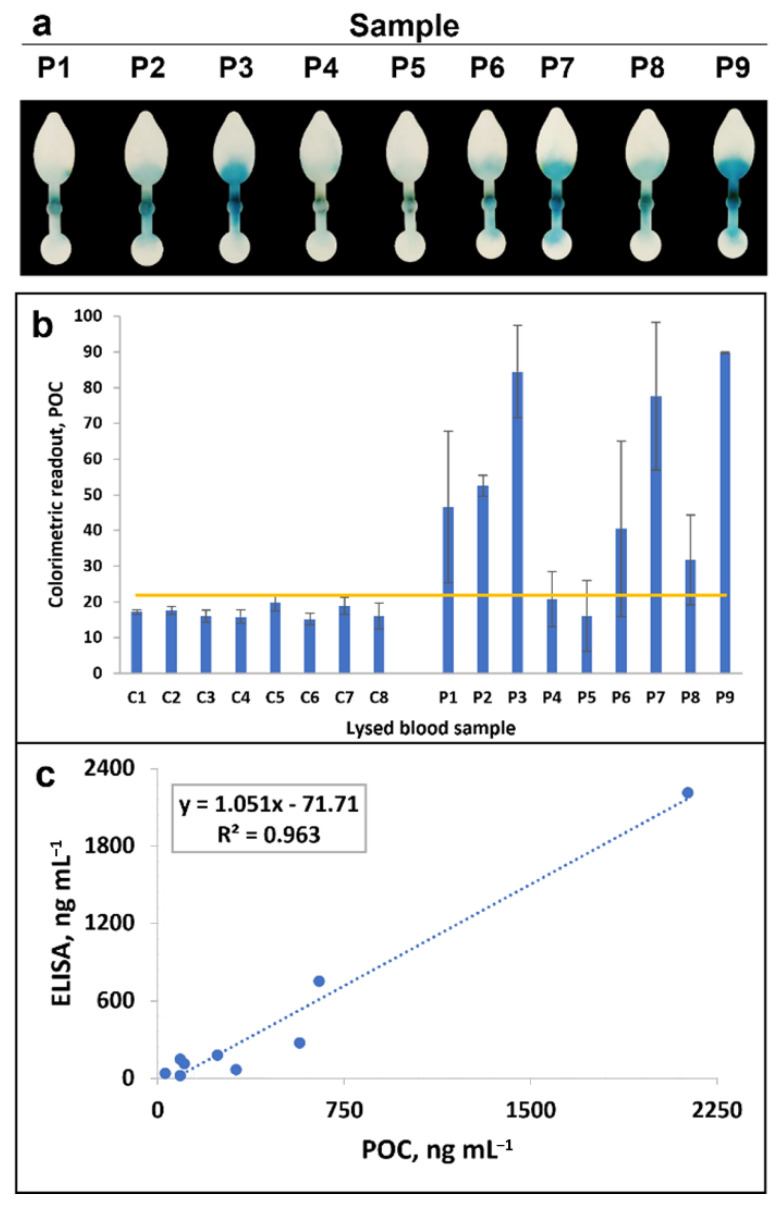
Detection of clinical whole blood samples using the µPAD. (**a**) Images of the colour readouts obtained in the µPAD for 9 blood samples obtained from patients infected with *P. falciparum* (lysed and diluted 1:10 with PBST-BSA). (**b**) Colorimetric readout obtained in samples negative (C1–8) or positive (P1–9) for *P. falciparum* (the horizontal line in the graph represents the analytical LOD, calculated from the signals and SD registered for the negative clinical samples). (**c**) Correlation of the concentration of Pf-LDH detected by the smartphone-based µPAD and the reference ELISA (samples diluted 1:10, except for sample P7, which was diluted to 1:100 due to signal saturation in the 1:10 dilution).

The µPAD was finally employed to study a battery of whole blood samples (diluted 1:10 and 1:100), 9 from patients infected with *P. falciparum* and 8 from individuals either healthy or displaying infections unrelated to malaria (Figure 5a and Figure S8). Samples were studied in parallel by standard microscopy and/or PCR at Vall d’Hebron Hospital and were also analysed by ELISA and one of the commercial RDT accredited by the WHO (Figure S9, Tables S1 and S2).

All the negative control samples displayed colour readouts that were below the POC LOD (Figure 5b). In contrast, 7 of the 9 malaria samples were clearly positive, exhibiting colour readouts above the test LOD, while the remaining two, which corresponded to submicroscopic malarias (negative by microscopy and confirmed by PCR and ELISA), displayed faint positives or negatives depending on the replicate. In positive samples, colour intensity visual interpretation allowed semi-quantitative result determination, with result agreement of 80–91% among untrained individuals (Figure S10). Furthermore, the concentration of Pf-LDH detected using the smartphone-based µPAD correlated with that obtained by ELISA (Figure 5c), providing quantitative results faster and with less handling.

Lastly, the samples were analysed using a commercial RDT, which detected in parallel Pf-HRP2 and pLDH. Although most commercial RDTs afford LODs in the range of 0.4–1.6 ng mL^−1^ when detecting Pf-HRP2, the few tests that detect pLDH display LODs spanning 10–1000 ng mL^−1^ [30]. This difference has also been noticed by several authors. For example, the multiplexed ELISA reported by Jang et al. exploited Quansys Q-Plex technology and exhibited LODs of 2.3, 47.8, and 75.1 pg mL^−1^ for HRP2, pLDH and *P. vivax* LDH (Pv-LDH), respectively [31]. The Luminex fluorescent assay developed by Martiáñez-Vendrell and co-workers detected HRP2, Pf-LDH and Pv-LDH with LODs of 6, 56 and 1093 pg mL^−1^ [32], respectively. Additionally, the multiplexed µPAD produced by Deraney and co-workers detected Pf-HRP2 and pLDH with LODs of 20.3 ng mL^−1^ and 80.2 ng mL^−1^, respectively [25]. Here, of the 9 malaria samples tested with the RDT, only 2 provided faintly positive test lines for pLDH, while the HRP2 result was positive in 7 and negative (P5) or faintly positive (P1) in the other 2 (Figure S9).

In summary, the µPAD developed here achieved remarkable results when tested against a commercial RDT, detecting malaria samples better than the RDT pLDH test and similarly to the Pf-HRP2 test. Furthermore, the device provided quantitative readouts that correlated with those obtained using standard methods (Table S1). Despite the small number of samples studied, these results show that the technology developed provides fast quantitative results, entailing little user intervention and a cost per test below 0.5 € (Table S3).

## 4. Conclusions

A μPAD has been developed that provides the smooth operation of single-step magneto-immunoassays, including MB magnetic concentration, washing and incubation with a chromogenic enzyme substrate. As has been shown, visual inspection of the colorimetric readout provides a semi-quantitative interpretation, while image capture and analysis using a smartphone camera and ImageJ deliver analyte quantification.

As a proof-of-concept, the system has been employed to detect Pf-LDH, a biomarker of malaria infection. Malaria antibody-based RDTs display turnaround times of 15–30 min and prices in the range of (1–5 €), delivering qualitative results. The μPAD developed in this work afforded quantitative Pf-LDH detection in lysed blood diluted 1:10 in ˂20 min (including 5 min of sample lysis), with LODs of 6.25 ng·mL^−1^ and 1.4 ng·mL^−1^ when interpreted by the naked eye and imaging analysis, respectively. Considering 1 parasite µL^−1^ equivalent to 0.06–0.2 ng·mL^−1^, these LODs correspond to 7–23 and 31–104 parasites µL^−1^, which is below the cut-off of 200 parasites µL^−1^ recommended by the WHO for RDTs. Furthermore, Pf-LDH quantitation provided by the μPAD correlated with that granted by the reference ELISA, but the μPAD was faster and easier to use.

Using the paper device to automate a magneto-immunoassay, rather than incorporating the biocomponents on-chip, converts the system into a versatile universal platform, easier to tune than classical RDTs by just changing the analyte in the single-step magneto-immunoassay step. With a low estimated production cost and result interpretation using a common smartphone, this μPAD is a versatile platform that could facilitate cost-effective POC testing in remote and low-resource settings.

## Supplementary Materials

The following supporting information can be downloaded at: https://www.mdpi.com/article/10.3390/bios12090680/s1, Figure S1: Optimization home-made light controlled box; Figure S2: Comparison of the single-step magneto-immunoassay developed in this work versus the assay previously developed; Figure S3: Geometrical evolution, washing strategy and signal intensity in the negative controls; Figure S4: Optimization of dispensing position and amount of TMB used in the μPAD for magneto-immunoassay detection; Figure S5: Optimization of the concentration of MBs in the paper-based magneto-immunoassay; Figure S6: Comparative performance of MBs from Invitrogen and GE Healthcare; Figure S7: Detection of Pf-LDH in PBST-BSA and blood using the POC device; Figure S8: Study of clinical samples using the POC device; Figure S9: Study of clinical samples using a commercial RDT; Figure S10: External evaluation of semiquantitative naked eye colour scale; Table S1: Summary of the results obtained in the analysis of clinical samples from malaria patients; Table S2: Summary of the results obtained for the control blood samples; Table S3: Estimate of the production cost of the retail-purchased reagents of μPAD.
