# Supplementary material for: Magnetic Bead Handling Using a Paper-Based Device for Quantitative Point-of-Care Testing
Source: Biosensors (Basel). 2022 Aug 25;12(9):680. doi: 10.3390/bios12090680 (PMC9496280; doi:10.3390/bios12090680)
Supplement: Supplementary file 1 [file biosensors-12-00680-s001.zip › biosensors-1859998-supplementary.pdf]

Magnetic bead handling using a paper-based device for quantitative point-of-care testing

Supplementary Material

Kevin Arias-Alpízar [1,2], Ana Sánchez-Cano [1,2], Judit Prat-Trunas [1], Elena Sulleiro [2,3,4], Pau Bosch-Nicolau [3,4], Fernando Salvador [3,4], Inés Oliveira [3,4], Israel Molina [3,4], Adrián Sánchez-Montalvá [2,3,4] and Eva Baldrich [1,4,*]

[1] *Diagnostic Nanotools Group, Vall d'Hebron Institut de Recerca (VHIR), 08035 Barcelona, Spain*

[2] *Universitat Autònoma de Barcelona (UAB), 08193 Bellaterra (Barcelona), Spain*

[3] *International Health Unit Vall d'Hebron-Drassanes, Vall d'Hebron Hospital Universitari, PROSICS Barcelona, 08035 Barcelona, Spain*

[4] *Centro de Investigación Biomédica en Red de Enfermedades Infecciosas (CIBERINFEC), Instituto de Salud Carlos III, 28028 Madrid, Spain*

***** Correspondence: eva.baldrich@vhir.org

Table of contents:

Material and methods.

Production of biotinylated detection antibodies (bd-Ab).

MB modification with c-MAb using EDC.

Results and discussion.

Figure S1. Optimization home-made light controlled box.

Figure S2. Comparison of the single-step magneto-immunoassay developed in this work *versus* the assay previously developed.

Figure S3. Geometrical evolution, washing strategy and signal intensity in the negative controls.

Figure S4. Optimization of dispensing position and amount of TMB used in the μPAD for magneto-immunoassay detection.

Figure S5. Optimization of the concentration of MBs in the paper-based magneto-immunoassay.

Figure S6. Comparative performance of MBs from Invitrogen and GE Healthcare.

Figure S7. Detection of Pf-LDH in PBST-BSA and blood using the POC device.

Figure S8. Study of clinical samples using the POC device.

Figure S9. Study of clinical samples using a commercial RDT.

Figure S10. External evaluation of semiquantitative naked eye colour scale.

Table S1. Summary of the results obtained in the analysis of clinical samples from malaria patients.

Table S2. Summary of the results obtained for the control blood samples.

Table S3. Estimate of the production cost of the retail-purchased reagents of μPAD (indirect and personnel costs not included).

Material and Methods

Production of biotinylated detection antibodies (bd-MAb).

The detection antibody (d-MAb) was first submitted to a buffer interchange in order to remove interfering reagents. For this, 300 µg of d-MAb were placed in an Amicon® Ultra 0.5 mL Centrifugal Filter (Merck Life Science, Madrid, Spain) in a final volume of 0.5 mL. The device was centrifuged at 14000 x g for 10 min, was filled with 0.5 mL of sodium carbonate buffer (0.1 M, pH 9.5), and was centrifuged again. This procedure was repeated once more. The concentrated d-MAb was then recovered, and sodium carbonate buffer was added to bring it to the initial concentration of 4.1 mg·mL^{-1}.

A biotin-XX, SSE (6-((6-((Biotinoyl)Amino)Hexanoyl)amino)Hexanoic Acid, Sulfosuccinimidyl Ester, Sodium Salt; Ref. B6352, Thermo Fisher Scientific; Barcelona, Spain) stock was prepared at a concentration of 2.5 mg·mL^{-1} in Milli-Q water and 9.66 µL were added to the d-MAb. The mixture was stirred at 24°C in the dark for 2 h. The biotin not bound to the d-MAb was eliminated using a PD G25 exclusion column (GE Healthcare, now Cytiva Europe, Freiburg, Germany) following the provider's instructions. The obtained bd-MAb was finally diluted to a concentration of 150 µg·mL^{-1} in 1% BSA and was stored at -20 °C.

MB modification with c-MAb using EDC

MB (1 mg in 100 µL) were washed twice with 15 mM MES using a magnetic separator (BILATEST, Merk). MB were next agitated for 15 min with 25 µg of c-MAb in 100 µL of 2 mg·mL^{-1} EDC in a thermoshaker (950 rpm; Thermal Shake lite; VWR International, Leuven, Belgium). After that, MB were serially washed with 200 µL of MES and PBS, and were blocked for 1 h with PBS, BSA 1%. The c-MAb-MB were then washed twice for 5 min with 100 µL of PBS, Tween 20 0.1% and were resuspended in 500 µL of PBS, Tween 20 0.1%, BSA 0.2% for storage at 4 °C (1.4-2.4×10^{9} MB·mL^{-1}, equivalent to 2 mg·mL^{-1}). The following figure illustrates the chemical reaction involved.

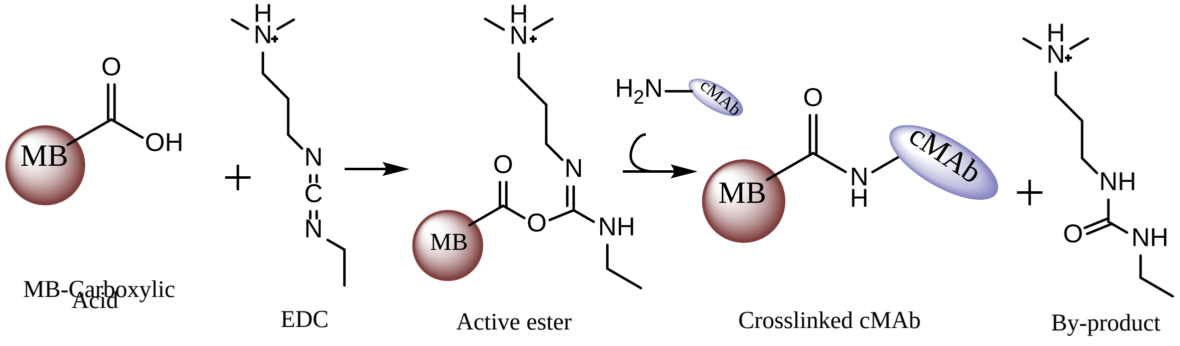

Figures

Figure S1. **Home-made dark box with controlled lighting.** (Left) A dark box was produced for image acquisition using a cardboard box (28cmx16cm x10cm), modified with a strip of LED lights (cool white 6000K LED dimmable strip lights with a power of 1200 lm) used for lighting the chamber. (Right) A smartphone was placed on top of the lid, which had a hole for the phone camera. The photograph shows the dark box placed upside-down.

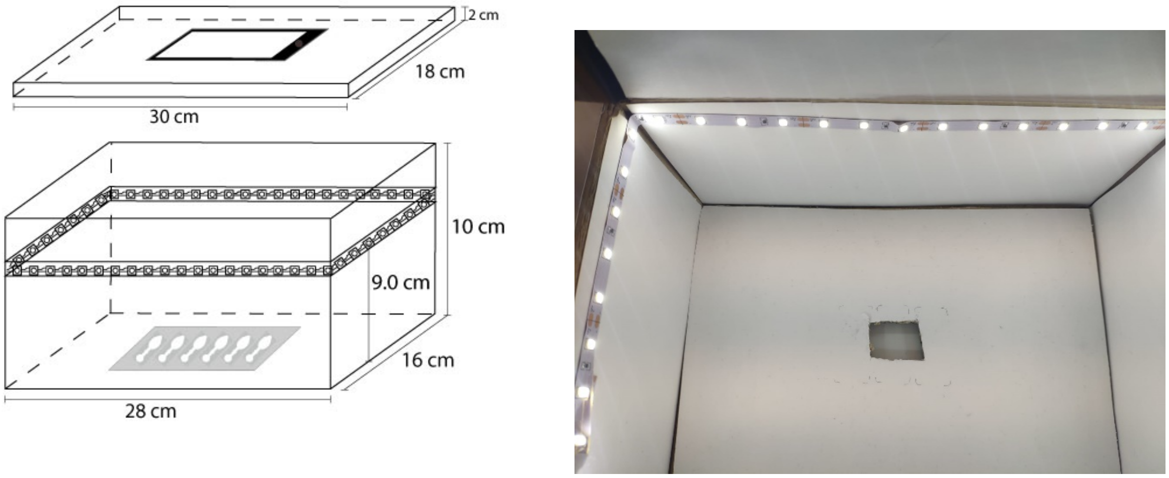

Figure S2. Comparison of the single-step magneto-immunoassay developed in this work *versus* the assay previously developed.

The starting point for this work was a single-step magneto-immunoassay developed previously for Pf-LDH detection, with some improvement [1,2]. That magneto-immunoassay relied on a customized nanoconjugate, produced by incubating bd-MAb and polyHRP (bd-MAb-polyHRP). A single-step magneto-immunoassay was next carried, which consisted in a single 5-min incubation of the sample (diluted with 1×RD to a final volume of 95 µL) with c-MAb-MB (4 µL) and the bd-MAb/Poly-HRP conjugate (0.5 µL; final concentration of bd-MAb and Poly-HRP of 225 ng mL^{-1} and 50 ng mL^{-1}, respectively). MB were then washed twice with 150 µL of PBST, and were stirred for 20 min in 100 µL of TMB substrate solution. After this, MBs were concentrated, the supernatant was transferred to 96-well plates, 50 µL of 1 M sulphuric acid were added to each well, and colorimetric detection was carried at 450 nm using a Sunrise plate reader (Tecan Group, Männedorf, Switzerland).

Although both bd-MAb and Poly-HRP were storage-stable, the bd-MAb/Poly-HRP conjugate was stable for a just month. In addition, the assay displayed a narrow linear range, with signal saturation above 12.5 ng mL^{-1} of Pf-LDH.

To overcome these drawbacks, here we used an optimized magneto-immunoassay (Figure 2a in the main manuscript), in which samples were incubated for 5 min with a cocktail of three reagents: c-MAb-MB (4 µL), bd-MAb (75 ng mL^{-1}) and Poly-HRP (50 ng mL^{-1}). As it can be observed in the graphs, this new assay displays similar signals (a) and signal-to-noise ratios (S/N; b) than the previous one for low-to-mid Pf-LDH concentrations, but higher signals and S/N for Pf-LDH concentrations ranging 10-50 ng mL^{-1} and wider linear range.

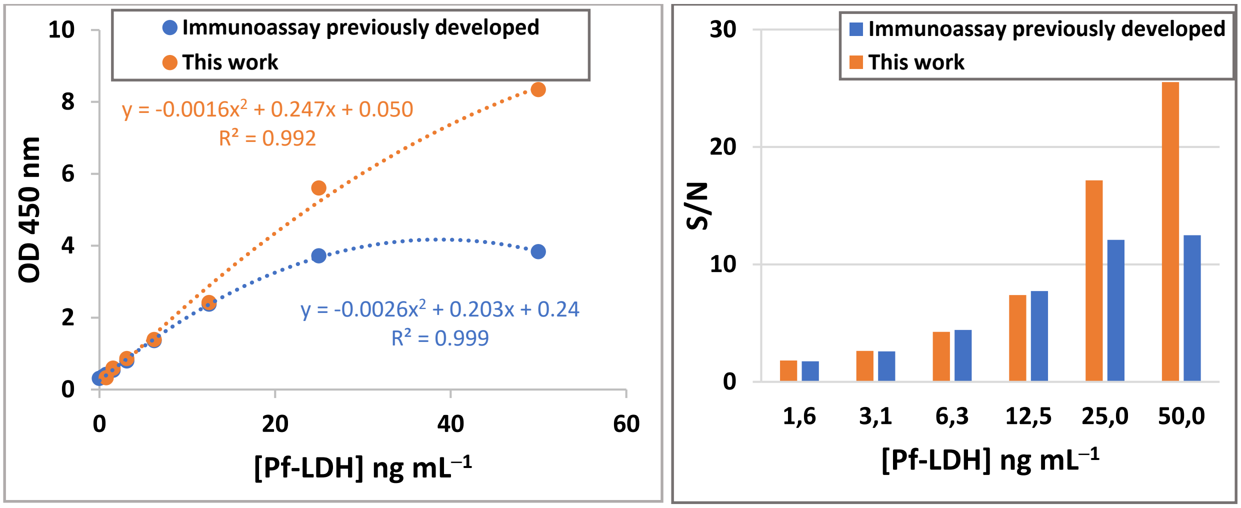

Figure S3. Geometrical evolution, washing strategy and signal intensity in the negative controls.

MB washing under flow conditions was optimized by mixing the MBs with 100 µL of a blue-stained aqueous solution. The mixture was then pipetted onto the washing pad of the devices. These had been placed onto a piece of acetate with a magnet for MB retention at the detection area, and with a pile of absorbent pads at the bottom. Serial additions of PBS 0.1× were then made, using alternatively 50, 100 or 200 µL of solution per wash. As it can be observed, for 50-µL additions, 5 consecutive washes were needed to remove completely the stained solution and observe the MBs concentrated in the central retention zone. For 100-200 µL washing volumes, 3-4 additions were enough. However, the addition 200 µL per washing volume saturated the paper sensor (sensor volume capacity ≈150 µL), which caused solution overflow, less efficient dye washing, and loss of MBs. In all the cases, and independently of the washing conditions, MPs were retained efficiently by the magnet in the detection area which could be seen by the naked eye.

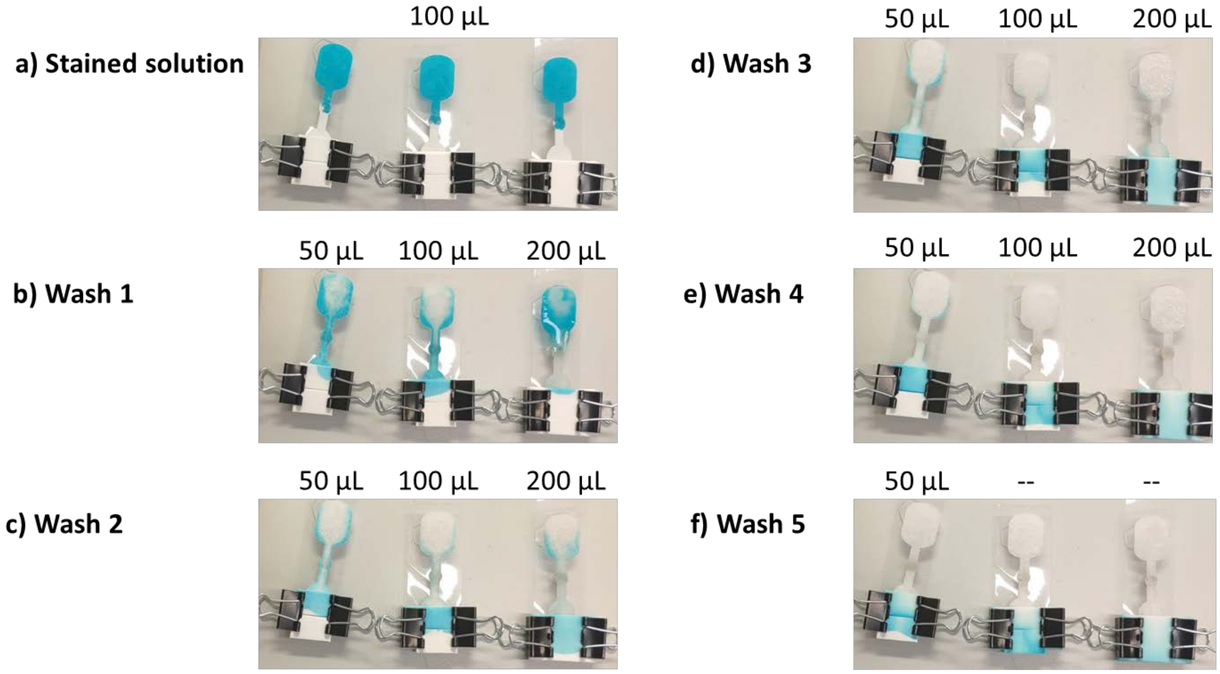

Figure S4. Optimization of dispensing position and amount of TMB used in the μPAD for magneto-immunoassay detection.

a) Examples of the colorimetric readouts obtained in the paper-based magneto-immunoassay for a positive control (25 ng·mL^{-1} of Pf-LDH) after dropping for detection 50 μL of TMB in three different positions. Dispensing the TMB directly onto the MBs (position 3) produced MB random rearrangement, decreasing signal intensity and reproducibility. Higher colour intensity and signal reproducibility were achieved if the substrate solution was dispensed upstream (positions 1 and 2). However, placing TMB too far from the MBs (i.e., position 1) facilitated TMB reflow towards the washing zone. This decrease TMB availability and colour evolution at the detection zone. Position 2 was chosen because it produced a more homogenous colour dispersion around the magnet and lower background signal in the blanks. b) Colorimetric readouts obtained for positive and negative controls (25 and 0 ng·mL^{-1} of Pf-LDH, respectively) when using for detection increasing volumes of TMB (dropped at position 2). A slight increment in the signals (both positive and negative) was observed when using increasing volumes of TMB, which was attributed to longer reaction times before substrate exhaustion occurred. The best signal-to-noise was achieved using 50 μL of TMB.

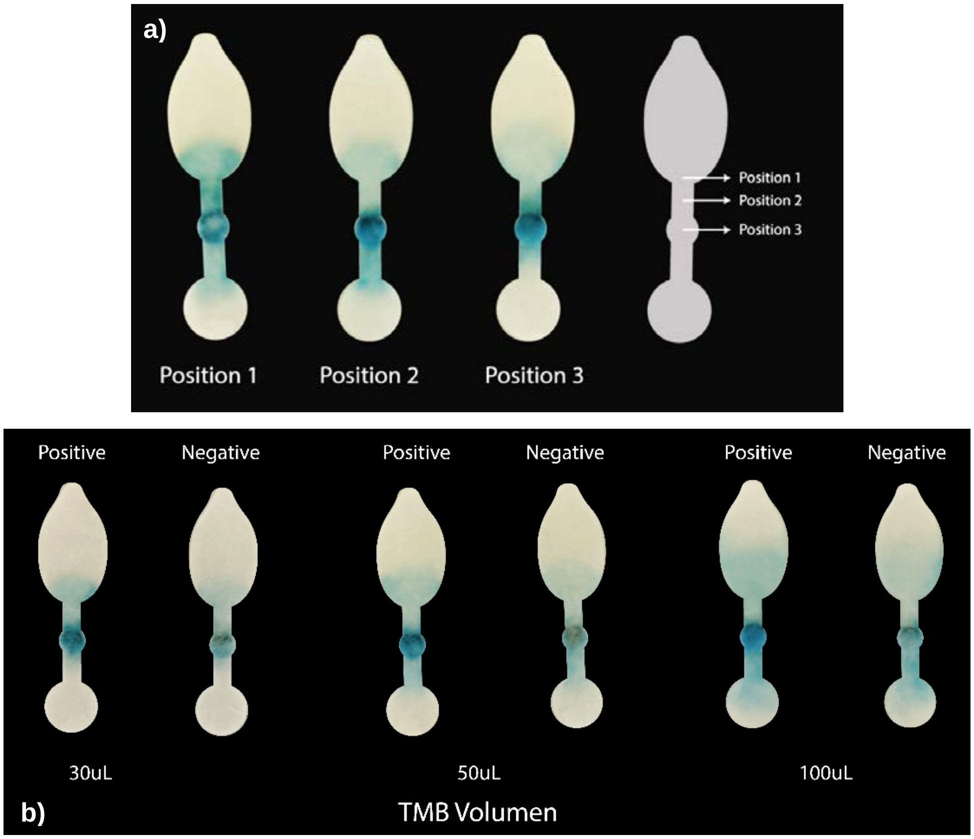

Figure S5. Optimization of the concentration of MBs in the paper-based magneto-immunoassay.

a) Colorimetric readouts (calculated using ImageJ) and b) S/N ratios obtained in the paper-based magneto-immunoassay for Pf-LDH concentrations ranging 12.5-50 ng·mL^{-1}, using for immunocapture three amounts of MBs (10-20 μg per sample; Invitrogen MyOne Dynabeads).

a)

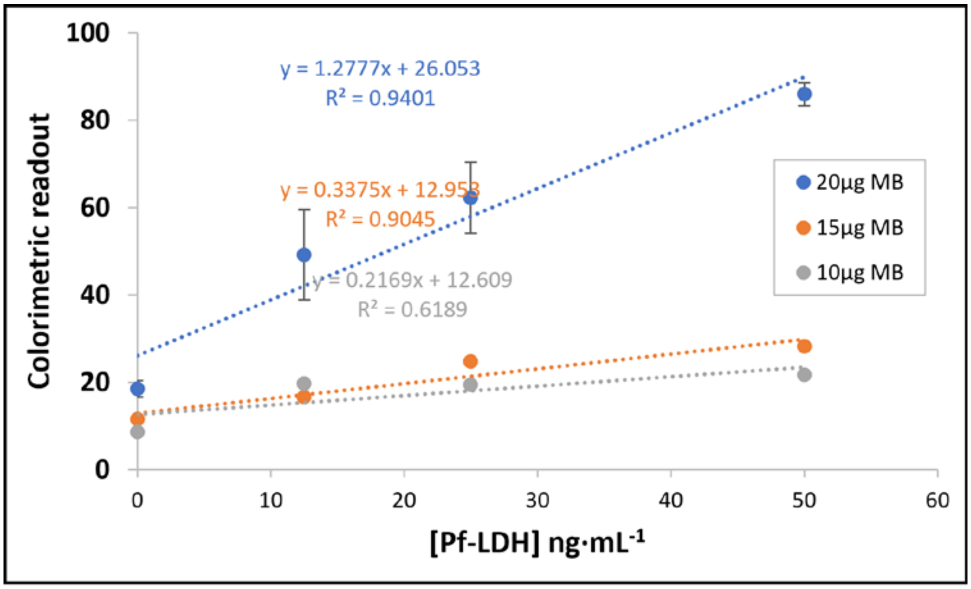

b)

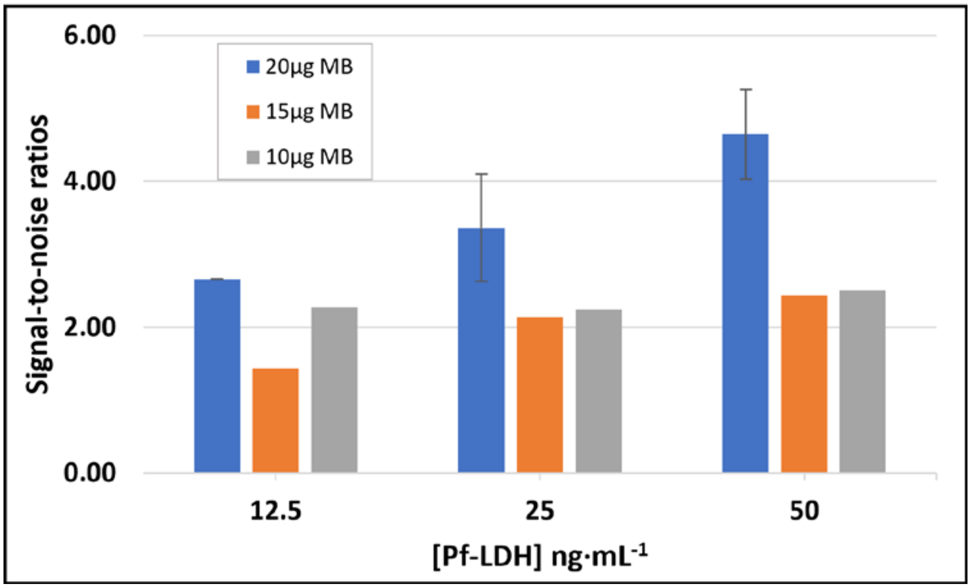

Figure S6. Comparative performance of MBs from Invitrogen and GE Healthcare. Invitrogen Dynabeads, used in the previous sections, were compared to GE Healthcare SeraMag MBs which, according to the provider, display higher magnetic content. In our experiments, SeraMag MBs exhibited faster magnetic concentration than Dynabeads. However, they provided also higher background noise and lower signals for all the concentrations of Pf-LDH studied, providing significantly lower S/N. (a) Optical densities measured at 450 nm for the magneto-immunoassay carried in tubes and (b) colorimetric readouts registered for the paper-based magneto-immunoassay for increasing Pf-LDH concentrations (1.56-12.5 ng·mL^{-1} and 3.13-50 ng·mL^{-1}, respectively), when using alternatively the two types of MBs (GE Healthcare SeraMag MBs and Invitrogen Dynabeads, in both cases carboxy-modified and 1 µm in diameter MBs, modified with c-MAb).

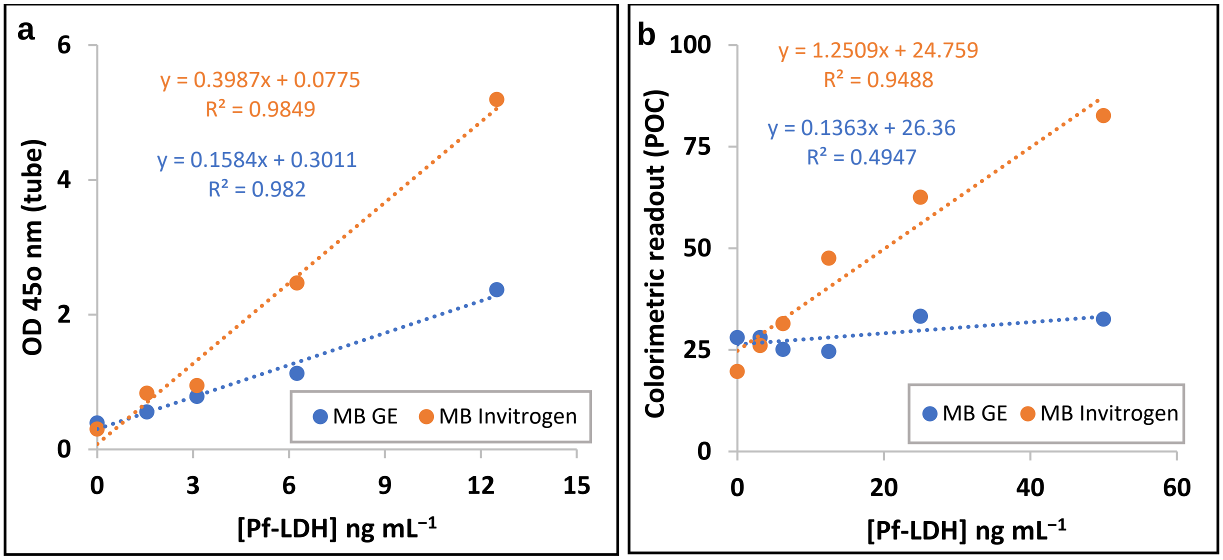

Figure S7. Detection of Pf-LDH in PBST-BSA and blood using the POC device.

Images obtained in four independent calibration experiments using the single-step magneto-immunoassay directly in the paper device to detect increasing concentrations of Pf-LDH in PBST-BSA or in lysed whole blood (diluted 1:10 and 1:100 with PBST-BSA). Each paper device was used only once. Accordingly, each image was obtained with a different paper device.

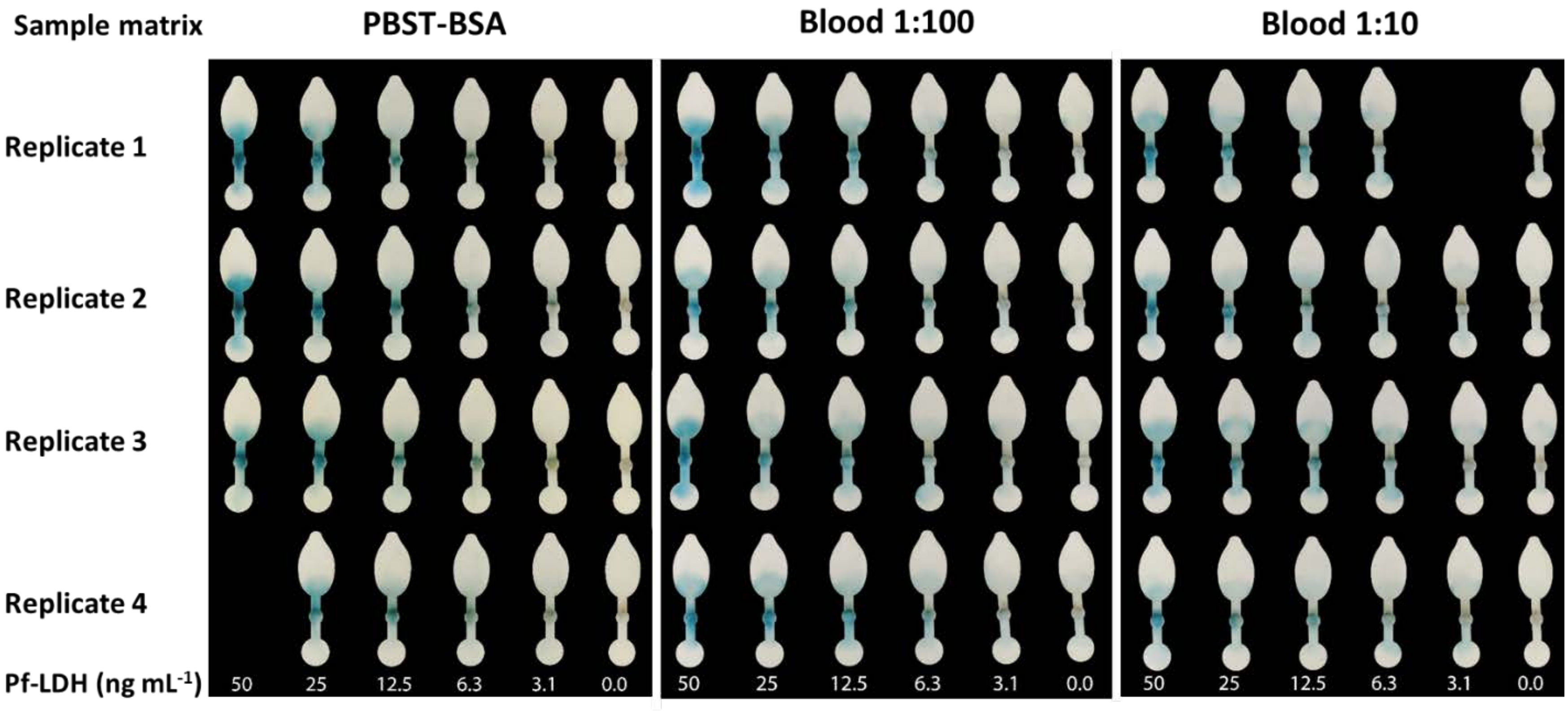

Figure S8. Study of clinical samples using the µPAD.

Nine whole blood samples obtained from malaria patients were analysed using the paper-based magneto-immunoassay. For this, samples were mixed 1:1 with lysis buffer, were incubated for 5 min and were diluted 1:10 and 1:100 with PBST-BSA. These lysed samples were then analysed using the paper-based magneto-immunoassay as detailed in the protocol in the main manuscript. Seven of the 9 samples were detected by the POC after a 1:10 dilution. Only samples P4 and P5, which corresponded to two submicroscopic malarias, were missed by the naked eye (one of the replicates was interpreted as a faint positive, but the other as negative). For samples diluted 1:100, only the highest parasitaemias could be detected (P3 and P9).

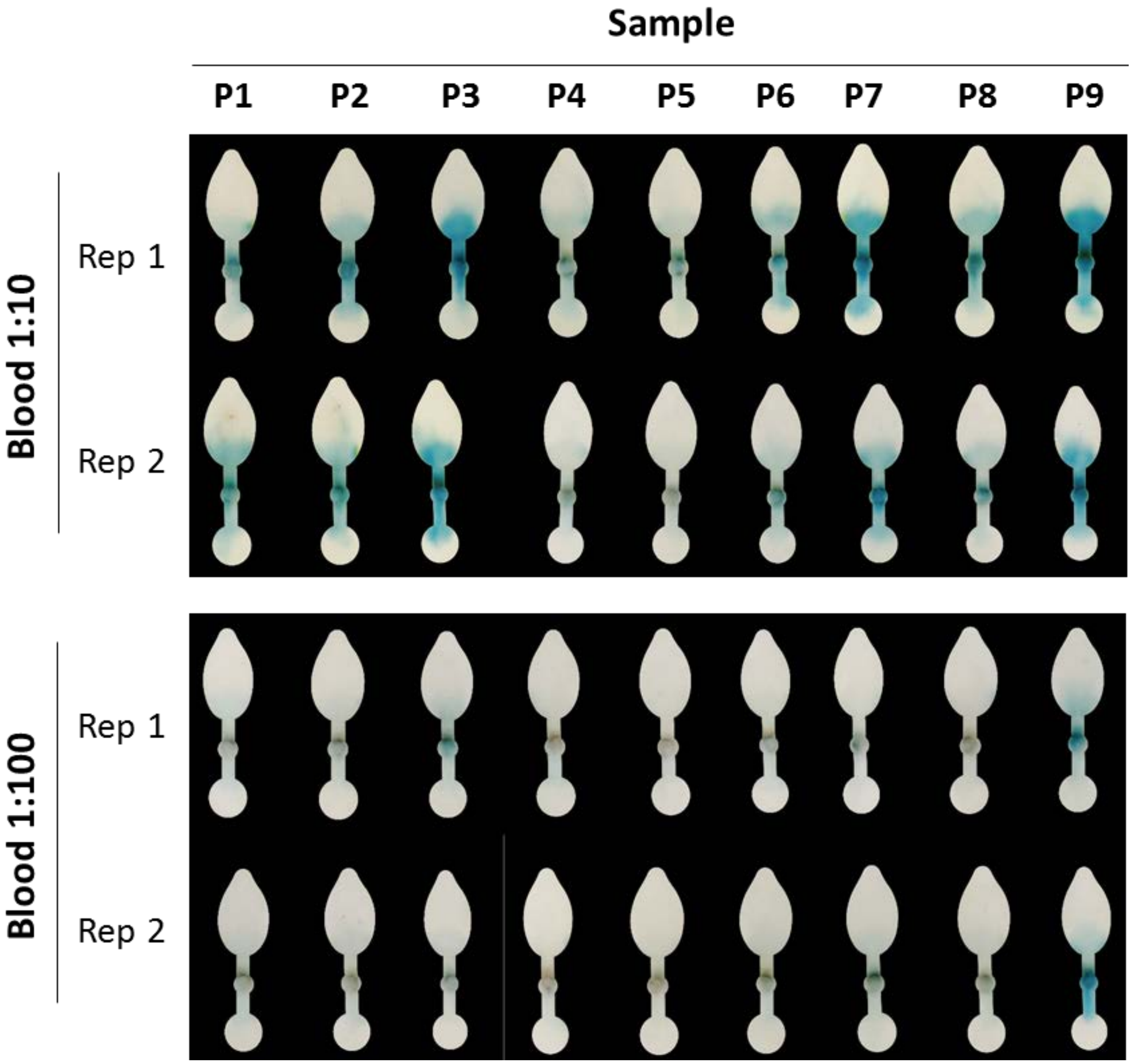

Figure S9. Study of clinical samples using a commercial RDT.

The nine clinical samples were analysed in parallel using a commercial RDT (SD BIOLINE Malaria Antigen Pf/Pan RDT, ABBOT - formerly Alere - ref. 5FK60), following the instructions provided by the supplier. This RDT displays a control line and two test lines for multiplexed detection of *Plasmodium falciparum* HRP2 (Pf-HRP2) and Pan *Plasmodium* LDH (pLDH).

Seven of the 9 samples were positive for Pf-HRP2 (P2, P3, P4, P6, P7, P8 and P9), P1 displayed a faint positive, and P5 was clearly negative for Pf-HRP2. In contrast, 7 of the samples were negative for pLDH and only two of the samples, P3 and P9, were faintly positive for pLDH.

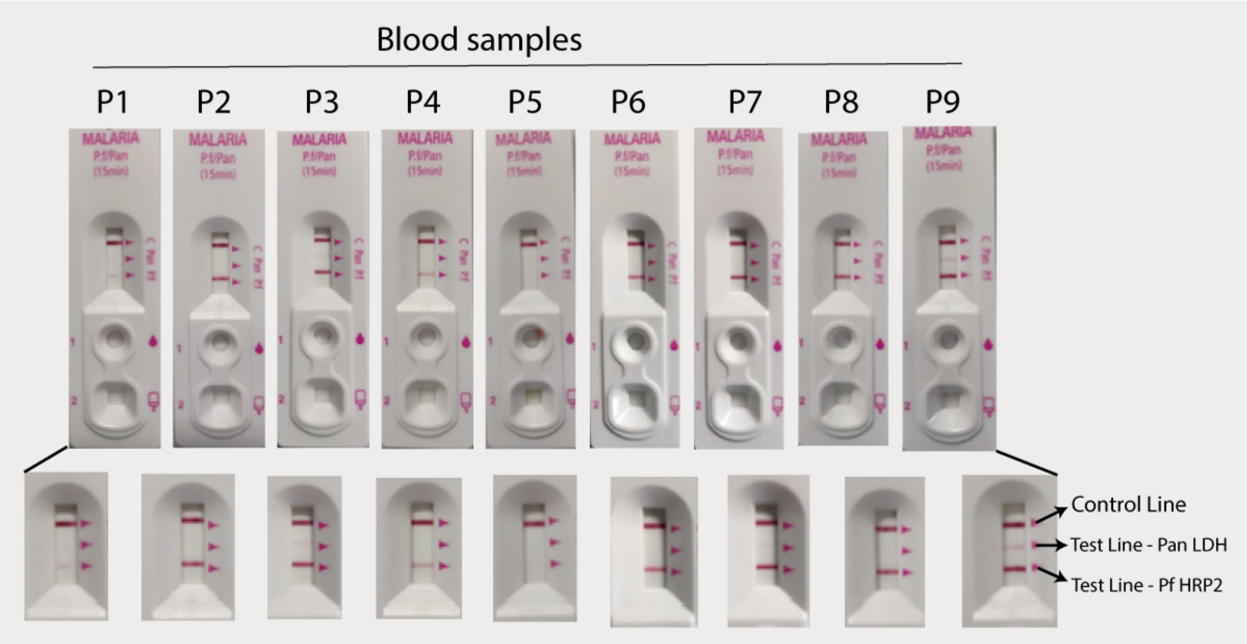

Figure S10. External evaluation of semiquantitative naked eye colour interpretation

Three questionnaires were produced to study the accuracy of µPAD result interpretation by users not familiar with the technology employed here. The forms were created using the EU Survey Tool for a correct data management in accordance with European legislation (EU) 2016/679. They were circulated in the social media and spread word of mouth among personal contacts and Vall d'Hebron Hospital personnel.

Each form included pictures of a calibrate and results obtained for a set of clinical samples (2 replicates each, but shown to users as independent samples). Users had to interpret colour intensity in the samples in a semi quantitative way, according to the corresponding colour reference scale of the calibrate.

The results obtained for each form are summarized next where, for each questionnaire, we show the pictures of the calibrate and samples that were offered to the volunteers. Result interpretation included here in the pictures of samples corresponds to the concentration of Pf-LDH obtained by ELISA and was not facilitated to users in the questionnaires. The graphs summarize the % of responses that attributed each quantitative category to each sample.

According to the results, parameters such as user sex, age and gender had no effect on result interpretation. In the same way, there were no statistical differences between the responses of individuals with and without previous experience in the performance and interpretation of rapid diagnostic tests, or between professionals working in the health sector and the rest. In contrast, result interpretation accuracy was slightly higher among individuals having responded using a computer screen, compared to those having used the screen of a mobile phone.

The **first form** used a reference colour code that included three categories (++, +, -) and the audience was asked to classify independently the two replicates of the nine malaria samples of Figure S-8 (blood 1:10). This form was answered by 136 individuals. As it can be observed, samples were interpreted with a high level of agreement, except for the first replicate of samples P4 and P5, which were close to the device limit of detection. Nevertheless, answers achieved a success rates of 84% and 89% for the first and second replicate sets, respectively.

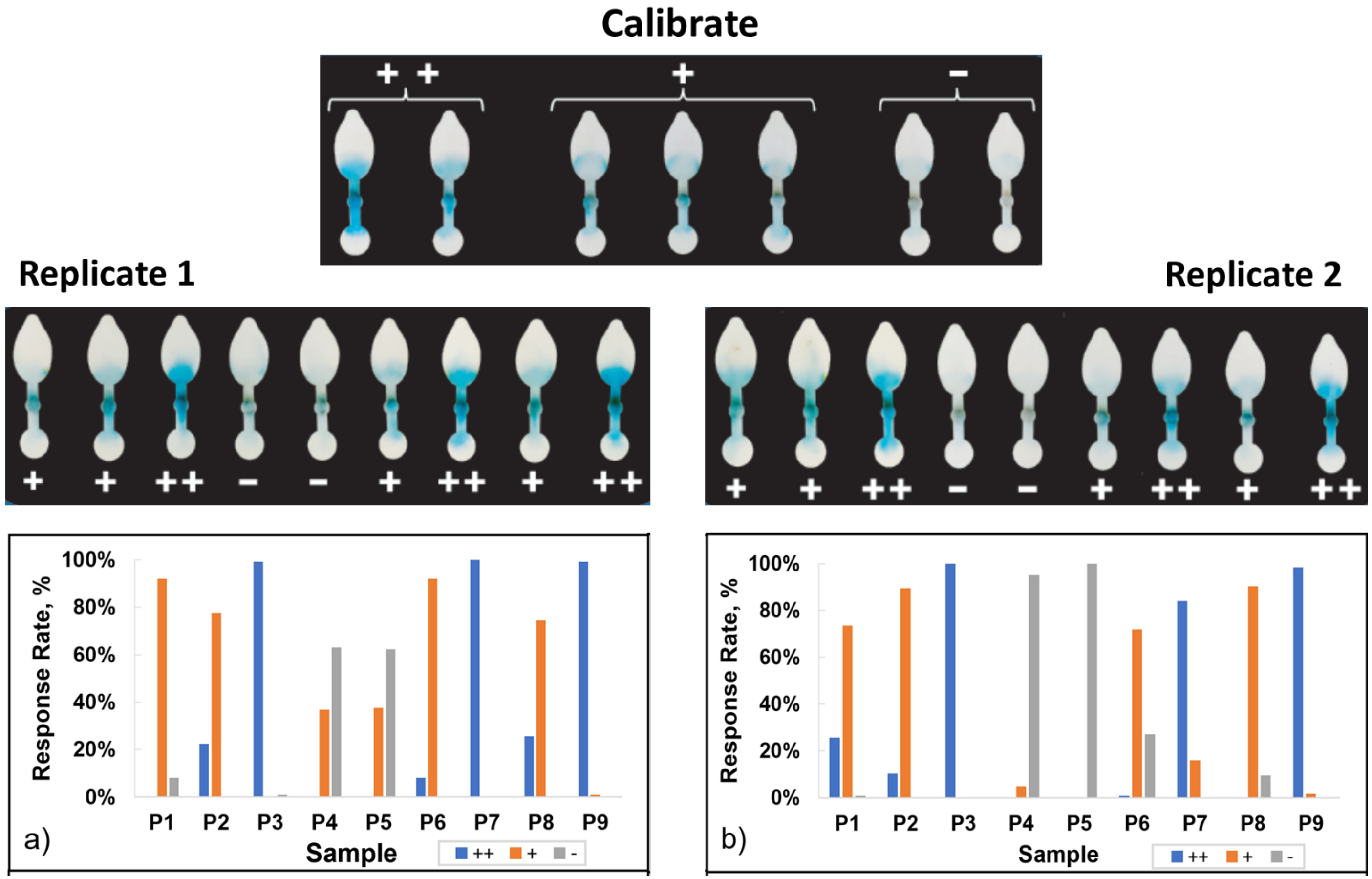

The **second form** used the same reference scale as the first form (++, +, -), but three negative samples were included after the images of the samples positive for malaria. This made that part of the interviewees (n=270) interpreted the first replicates of samples P4 and P5 and positives, with average success rates of 80% and 90% for the first and second replicate sets, respectively.

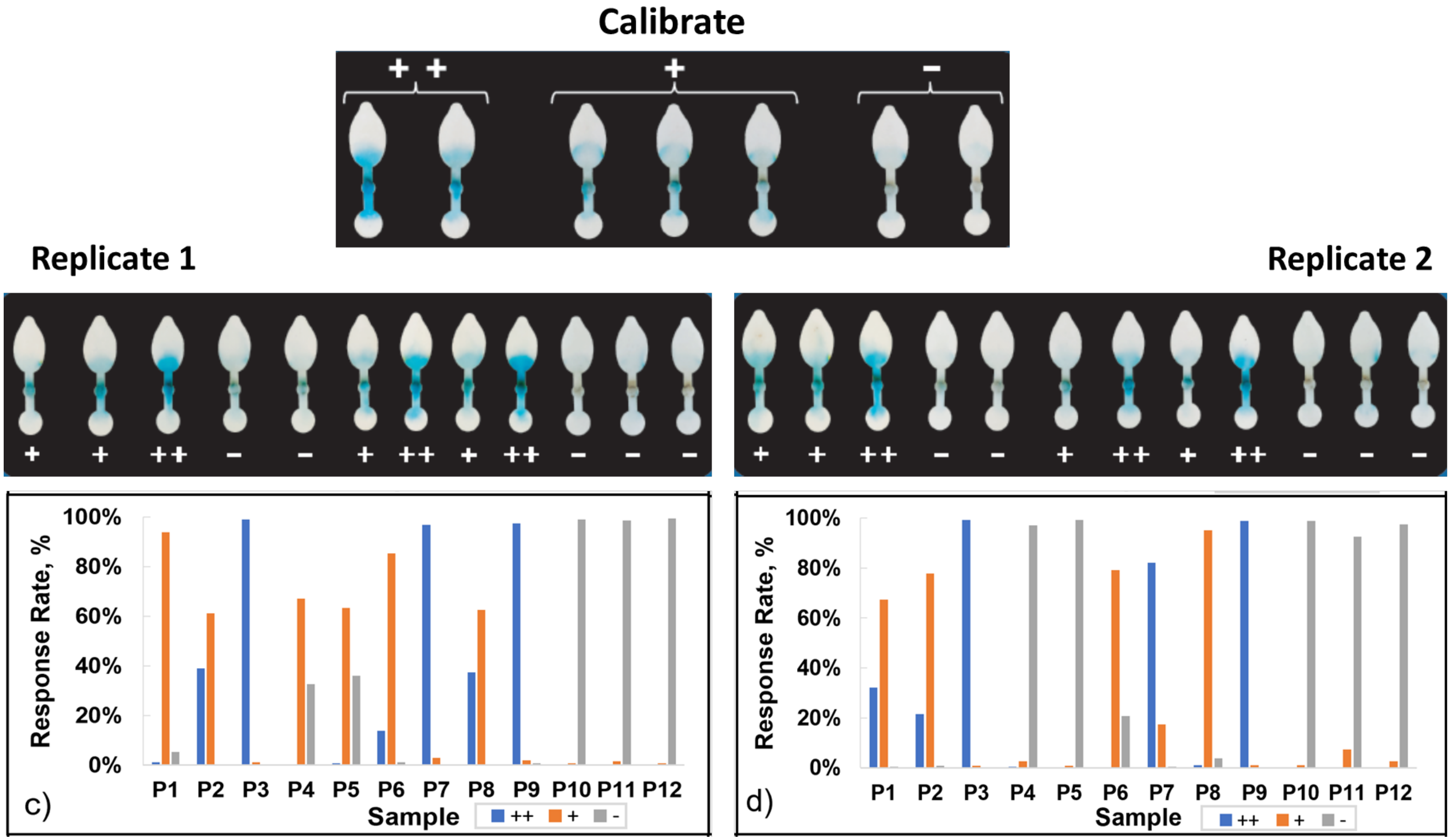

Finally, in the **third form**, a new category was added to the reference scale (+++, ++, +, -) in an attempt to obtain a semi quantitative system (for upper-, middle- and low-parasitaemia malaria positive samples). In this last form, 9 positive and 3 negative outcomes were given to the public, whose replicates were reassembled randomly in the two pictures of samples. Contrary to the first two forms, in this case instructions were given to facilitate result interpretation. The 77 users who answered had success rates of 91% and 82% in the first and second replicate sets, respectively.

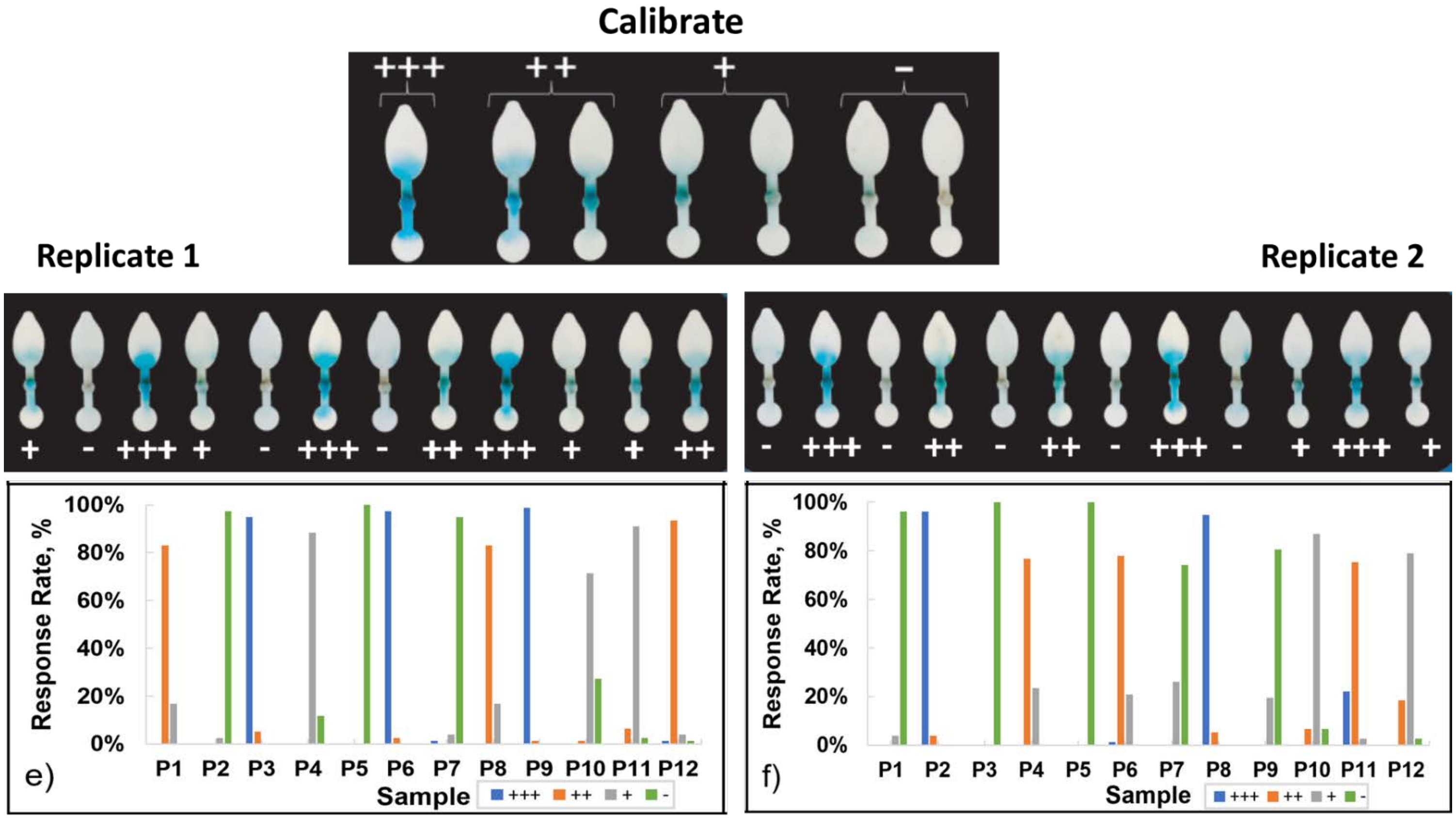

Table S1. Summary of the results obtained in the analysis of clinical samples from malaria patients.

Blood samples from 9 patients infected with *P. falciparum* were analysed by microscopy, ELISA, a commercial RDT (detecting both biomarkers Pf-HRP2 and Pan LDH), and the µPAD developed in this work. Visual interpretation of the µPAD readout was made independently on the two replicates obtained per sample (Figure S-8; blood 1:10), taking as the reference the calibrate in Figure 4a in the main manuscript. The concentration of Pf-LDH was calculated by interpolation of the images' analysis in the calibrate obtained for spiked blood (1:10; Figure 4c).

Sample	Microscopy (% parasitaemia)	ELISA ($ng\ mL^{-1}$)	Commercial RDT		µPAD		
			Pf-HRP	Pan LDH	Rep 1	Rep 2	$ng\ mL^{-1}$
**P1**	+ (0.3)	+ (91)	-/+	-	+	+	145
**P2**	+ (0.6)	+ (240)	+	-	+	++	177
**P3**	+ (0.3)	+ (649)	+	+	++	++	750
**P4**	-	+ (30)	+	-	+/-	-	36
**P5**	-	+ (91)	-	-	-	-	19
**P6**	+ (0.2)	+ (106)	+	-	+	+	112
**P7**	+ (0.1)	+ (570)	+	-	++	++	274
**P8**	+ (0.8)	+ (316)	+	-	+	+	65
**P9**	+ (0.8)	+ (2132)	+	+	++	++	2212

Table S2. Summary of the results obtained for the control blood samples.

Blood samples from 8 healthy patients were analysed by ELISA, a commercial RDT (detecting both biomarkers Pf-HRP2 and Pan LDH), and the μPAD developed in this work. Visual interpretation of the μPAD readout was made independently on the two replicates obtained per sample (blood 1:10), taking as the reference the calibrate in Figure 4a in the main manuscript.

Sample	ELISA	Comercial RDT		μPAD
		Pf-HRP	Pan LDH	
C1	-	-	-	-
C2	-	-	-	-
C3	-	-	-	-
C4	-	-	-	-
C5	-	-	-	-
C6	-	-	-	-
C7	-	-	-	-
C8	-	-	-	-

Table S3. Estimate of the production cost of the retail-purchased reagents of µPAD (indirect and personnel costs not included).

Reagent	Amount / test	Price / stock unit	Price / test
MBs	20 µg	144.40 € / 20 mg	0.144
c-MAb	0.5 µg	165.60 € / 1 mg	0.0828
bd-MAb	8.25 ng	165.60 € / 1 mg	0.0014
Poly-HRP	5.5 ng	215.1 € / 0.5 mg (0.5 mg·mL^{-1})	0.0024
TMB	50 µL	237 € / 400 mL (4x100mL)	0.0296
Sensors (Standard 17)	1	8.72 € / sheet = 114 chips	0.0765
Absorbent pads (CF5)	1	7.99 € / sheet = 72 absorbent pads	0.1110
		**Total / test**	**0.4480**

Bibliography

1. Ruiz-Vega, G.; Arias-Alpízar, K.; de la Serna, E.; Borgheti-Cardoso, L. N.; Sulleiro, E.; Molina, I.; Fernàndez-Busquets, X.; Sánchez-Montalvá, A.; del Campo, F. J.; Baldrich, E. Electrochemical POC Device for Fast Malaria Quantitative Diagnosis in Whole Blood by Using Magnetic Beads, Poly-HRP and Microfluidic Paper Electrodes. *Biosens. Bioelectron.* **2020**, *150*, 111925, doi:10.1016/j.bios.2019.111925.
2. Arias-Alpízar, K.; Sánchez-Cano, A.; Prat-Trunas, J.; de la Serna, E.; Alonso, O.; Sulleiro, E.; Sánchez-Montalvá, A.; Diéguez, A.; Baldrich, E. Malaria quantitative POC testing using magnetic particles, a paper microfluidic device and a hand-held fluorescence reader. *Biosens. Bioelectron.* **2022**, *215*, 114513, doi.org/10.1016/j.bios.2022.114513.
